# A novel chemical, STF-083010, reverses tamoxifen-related drug resistance in breast cancer by inhibiting IRE1/XBP1

**DOI:** 10.18632/oncotarget.5827

**Published:** 2015-10-19

**Authors:** Jie Ming, Shengnan Ruan, Mengyi Wang, Dan Ye, Ningning Fan, Qingyu Meng, Bo Tian, Tao Huang

**Affiliations:** ^1^ Department of Breast and Thyroid Surgery, Union Hospital, Tongji Medical College, Huazhong University of Science and Technology, Wuhan 430022, P.R. China; ^2^ Department of Breast and Thyroid Surgery, Luoyang Central Hospital, Zhengzhou University, Luoyang, Henan 471009, P.R. China; ^3^ Department of Radiation Oncology, Peking Union Medical College hospital, Beijing 100730, P.R. China; ^4^ Department of Neurobiology, Tongji Medical College, Huazhong University of Science and Technology, Wuhan 430030, P.R. China; ^5^ Key Laboratory of Neurological Diseases of Hubei Province, Tongji Medical College, Huazhong University of Science and Technology, Wuhan 430030, P.R. China

**Keywords:** STF-080310, tamoxifen, breast cancer, IRE1/XBP1

## Abstract

Recent studies show that the unfolded protein response (UPR) within the endoplasmic reticulum is correlated with breast cancer drug resistance. In particular, human X-box binding protein-1(XBP1), a transcription factor which participates in UPR stress signaling, is reported to correlate with poor clinical responsiveness to tamoxifen. In this study, we develop a tamoxifen-resistant MCF-7 cell line by treating the cell line with low concentration of tamoxifen, and we find that XBP1 is indeed up-regulated at both the mRNA and protein levels compared to normal MCF-7 cells. STF-083010, a novel inhibitor which specifically blocks the XBP1 splicing, reestablishes tamoxifen sensitivity to resistant MCF-7 cells. Moreover, co-treatment with STF-083010 and tamoxifen can significantly delay breast cancer progression in a xenograft mammary tumor model. We next investigate the expression of XBP1s in over 170 breast cancer patients' samples and the results demonstrate that XBP1s expression level is highly correlated with overall survival in the ER^+^ subgroup, but not in the ER^−^ subgroup, suggesting a potential therapeutic application of XBP1 inhibitors in ER^+^breast cancer treatment.

## INTRODUCTION

Tamoxifen (TAM) is one of the most frequently used, and effective, endocrine treatment drugs, which can reduce mortality and breast cancer recurrence in patients with hormone receptor positive breast tumors. Unfortunately, up to 50% of estrogen or progesterone receptor positive breast cancers do not respond to endocrine therapies, displaying de novo or intrinsic resistance. Therefore, the development of novel and efficient therapies for tamoxifen-resistant breast cancer remains a major challenge for breast cancer researchers and clinicians. [1] The unfolded protein response (UPR), a collective set of signaling pathways which is activated by endoplasmic reticulum (ER) stress, has been demonstrated to be one of the most important endocrine treatment-resistant mechanisms and represents a potential therapeutic target for tamoxifen-resistant breast cancer. [2] However, the detailed mechanisms of how the three UPR downstream branches (IRE1, PERK, and ATF6) integrate their cyto-protective and proapoptotic outputs under ER stress, such as hypoxia, starving or tamoxifen treatment, are still unknown. [3] Some studies have suggested that the ER transmembrane kinase/endoribonuclease (RNase) IRE1alpha is a key component of the cell-fate switch for UPR-triggered apoptosis or survival [4, 5].

XBP1 is reported to be an important regulator of the UPR [5]. It has been shown that only the spliced form of XBP1 (XBP1s) can induce the UPR efficiently. Moreover, XBP1s is more stable, easier to transport and a stronger transcriptional factor compared to its unspliced form, XBP1u. [6]

Previous studies have demonstrated that XBP1 expression is increased in estrogen therapy resistant breast cancer cell lines and is co-expressed with the estrogen receptor alpha (ERalpha) in breast tumors. [7, 8] Overexpression of XBP1s in ER-positive breast cancer cells leads to estrogen-independent cell growth and reduced sensitivity to growth inhibition induced by tamoxifen and Faslodex independent of a functional p53. [9] The ratios of XBP1s/XBP1u mRNA (indicating enhanced splicing by IRE1) in 100 primary breast cancer patients who received tamoxifen treatment were measured by quantitative RT-PCR, and the result suggests that higher ratios of XBP1s/XBP1u are correlated with poorer survival [10]. Gene expression profile analysis reveals that XBP1s acts through transcriptional regulation of the estrogen receptor, the antiapoptotic gene BCL2, and several other genes associated with cell cycle and apoptosis. [11, 12]

XBP1s expression level may be one of the key players in tamoxifen-resistant breast cancer, while IRE1, the upstream nuclease which mediates the splicing of XBP1 pre-mRNA, may be a potential target for reducing resistance. Classical IRE1-XBP1 inhibitors, such as sunitinib and AYP29, not only inhibit the kinase function of IRE1but also activates its endonuclease activity, which has no effect on XBP1in terms of expression. [13] Intriguingly, a new type of IRE1-XBP1 inhibitor, STF-083010, inhibits only IRE1 RNase activity but does not alter the phosphorylation process, thus decreasing XBP1s protein level. [14]

In this study, we demonstrate increased levels of XBP1s both at the mRNA and protein level in a tamoxifen-resistant MCF-7 cell line (termed MCF7-TAMR hereafter) compared to normal MCF-7 cells (termed control cells hereafter). Moreover, we also find that STF-083010, a novel inhibitor which specifically blocks XBP1 splicing, can re-establish MCF7-TAMR cells' sensitivity to tamoxifen treatment *in vitro*. Furthermore, we demonstrate the synergistic effect of STF-083010 and tamoxifen in controlling breast cancer progression in a xenograft murine mammary cancer model. Finally, we investigate XBP1s expression in over 170 breast cancer patients' samples, and show that XBP1s expression is highly correlated with overall survival in ER^+^ breast cancer patients, strongly suggesting a potential therapeutic application of XBP1 inhibitors in breast cancer treatment.

## RESULTS

### Establishment of MCF7-TAMR and T47D-TAMR cell lines

**Figure 1:**
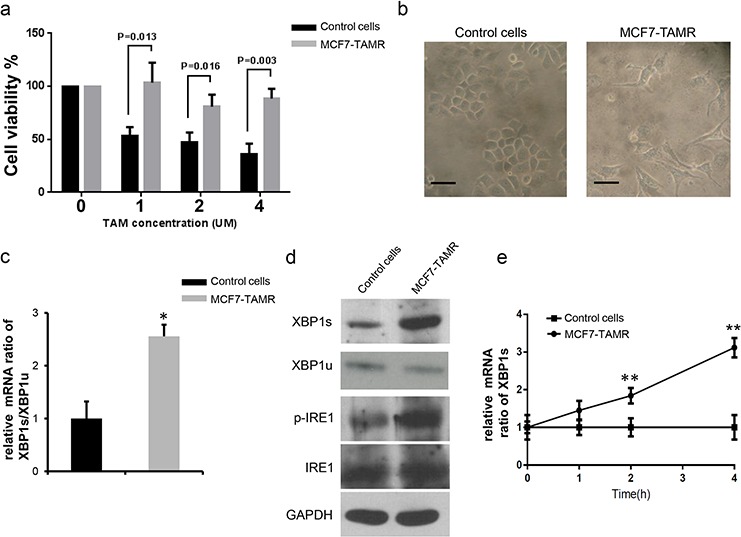
Elevated XBP1s expression level is associated with acquired tamoxifen resistance **a.** Long-term treatment (30days) with low tamoxifen concentration induced tamoxifen-resistance in MCF-7 cells, which showed significantly less sensitivity to tamoxifen under different concentrations. Cell viabilities were determined 48 hours after tamoxifen treatment by MTT assay at least 3 times at different conditions, mean survival (±s.e.m.) of control MCF-7 cells and MCF7-TAMR cells are shown in the bar graph. (unpaired, two-sided *t*-test). **b.** Morphological analysis of control and MCF7-TAMR cells by microscopy. Note the control cells exhibit round shape character, while resistant cells show more branches and flat morphology, which are more similar to normal breast epithelia. Scale bars, 100 μm. **c.** mRNA level of XBP1s/XBP1u ratio are determined by RT-PCR in both control and MCF7-TAMR cells. The ratio in MCF7-TAMR cells is significantly higher compared to control cells, suggesting a highly active splicing process in MCF7-TAMR cells. *P < 0.05. (unpaired, two-sided *t*-test). **d.** Immunoblot analysis of XBP1s and p-IRE1 protein levels in control and MCF7-TAMR cells. Representative results are shown. Note the XBP1s and phosphorylated IRE1expression level in MCF7-TAMR cells are significantly higher than control cells, indicating a more dynamic IRE1-XBP1 axis in MCF7-TAMR cells. The XBP1u, IRE1 and GAPDH are used as loading controls. **e.** Time dependent analysis of XBP1s mRNA level in MCF7-TAMR cells by RT-PCR. Results from three independent experiments are shown in mean ratio (±s.e.m). Note that the control cells mRNA level are always normalized to 1 in all conditions. *P < 0.05; **P < 0.01.(unpaired, two-sided *t*-test).

To develop a MCF7-TAMR cell line, we exposed normal MCF-7 cells to a low concentration of tamoxifen (1 μM) for 30 days continuously and then compared the viability of these treated cells to control cells at 1 μM, 2 μM and 4 μM tamoxifen. The results showed that the MCF7-TAMR cells exhibited significantly less sensitivity to tamoxifen treatment at all three concentrations compare to the control cells (Figure 1a). Microscopic analysis was used to assess the morphological changes upon tamoxifen treatment; we found that control MCF-7 cells displayed a highly round morphology, while the MCF7-TAMR cells showed more branches and displayed a long, flat morphology which were similar to normal tissue epithelia (Figure 1b). We also developed T47D-TAMR cells by exposing control T47D cells to 10 μM tamoxifen for 30 days continuously.

### Elevated XBP1s level in the MCF7-TAMR and T47D-TAMR cell lines

To compare XBP1 expression level between control cells and tamoxifen resistant cells, we performed RT-PCR to analyze the mRNA level of XBP1s and XBP1u, and the XBP1s/XBP1u ratio was used as a measure of XBP1 splicing activity. We found that both in MCF7-TAMR and T47D-TAMR cells, the XBP1s/XBP1u ratio was significantly higher than control cells (Figure 1c, Supplementary Figure S1b), suggesting more efficient XBP1 processing. Immunoblot analysis also showed that the XBP1s protein level was significantly higher in MCF7-TAMRcells and T47D-TAMR cells and in line with this, the XBP1 upstream regulator, IRE1, showed higher activity (increased phosphorylation) in MCF7-TAMR cells and T47D-TAMR cells (Figure 1d, Supplementary Figure S1c). We also investigated AKT and Caspase3 activities, but no significant difference was found between control cells and tamoxifen resistant cells. Interestingly, when we treated MCF7-TAMR cells with 4 μM tamoxifen and monitored the spliced XBP1s mRNA level, we found that the XBP1s mRNA was increased in a time dependent manner only in the MCF7-TAMRcells (Figure 1e), suggesting that XBP1's splicing was correlated with the tamoxifen treatment in a time dependent manner.

### The IRE1/XBP1 inhibitor, STF-080310, restores tamoxifen sensitivity in MCF7-TAMR cells

**Figure 2:**
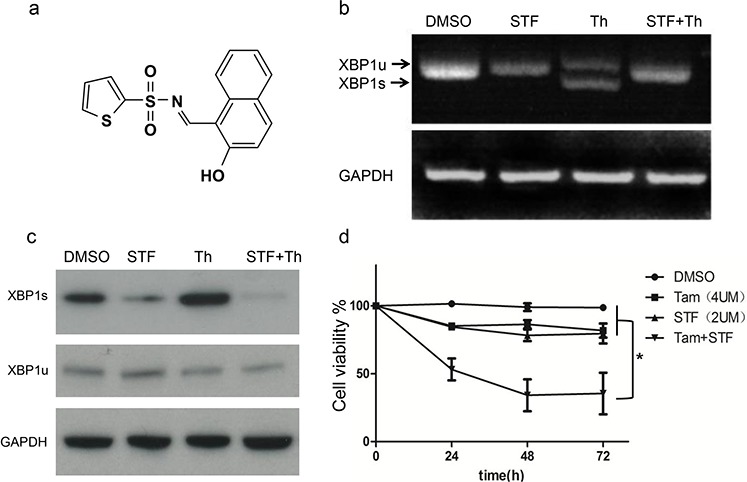
A novel IRE1/XBP1 inhibitor STF-080310 can block XBP1 splicing and restore tamoxifen sensitivity *in vitro* **a.** Schematic chemical structure of STF-080310 (STF). **b.** RT-PCR analysis of XBP1 splicing in MCF7-TAMR cells. Thapsigargin(Th) is a classical chemical which can trigger UPR and XBP1 splicing. The upper band indicates XBP1u mRNA and the lower band indicates XBP1s mRNA. Note that STF can efficiently block XBP1 mRNA splicing even in Th-treated cells. **c.** Immunoblot analysis of XBP1s in DMSO, STF, Th and STF plus Th treatment in MCF7-TAMR cells, The XBP1u, and GAPDH are used as loading controls. **d.** Cell viability assay of MCF7-TAMR cells treated with STF-080310, tamoxifen, and dual treatment of STF and tamoxifen. Results from three different experiments are shown and suggest that STF can significantly restore tamoxifen sensitivity *in vitro* by inhibiting XBP1s activity. *P < 0.05. (unpaired, two-sided *t*-test).

STF-080310 is a novel IRE1/XBP1 inhibitor which can specifically inhibit the RNase activity of IRE1without affecting its kinase function (Figure 2a), and therefore can block XBP1 splicing and decrease XBP1s levels. [14]. Thapsigargin is a classical molecule which can trigger endoplasmic reticulum (ER) stress and activate XBP1 splicing. When we treated MCF7-TAMR cells with thapsigargin, the XBP1 splicing was induced. However, when we co-applied STF-080310 with thapsigargin, the splicing process of XBP1 was efficiently blocked (Figure 2b, 2c), indicating that STF-080310 efficiently inhibits XBP1 function. Indeed, when we treated MCF7-TAMR cells with tamoxifen or STF-080310 separately, the cell viability was not affected, but when the MCF7-TAMRcells were treated with tamoxifen and STF-080310 together, the cell viability was significantly reduced (Figure 2d). Taken together, these results demonstrate that the IRE1/XBP1 inhibitor STF-080310 can restore tamoxifen sensitivity in MCF7-TAMR cells.

### STF-080310 suppresses breast tumor progression in a murine breast cancer xenograft model together with tamoxifen

**Figure 3:**
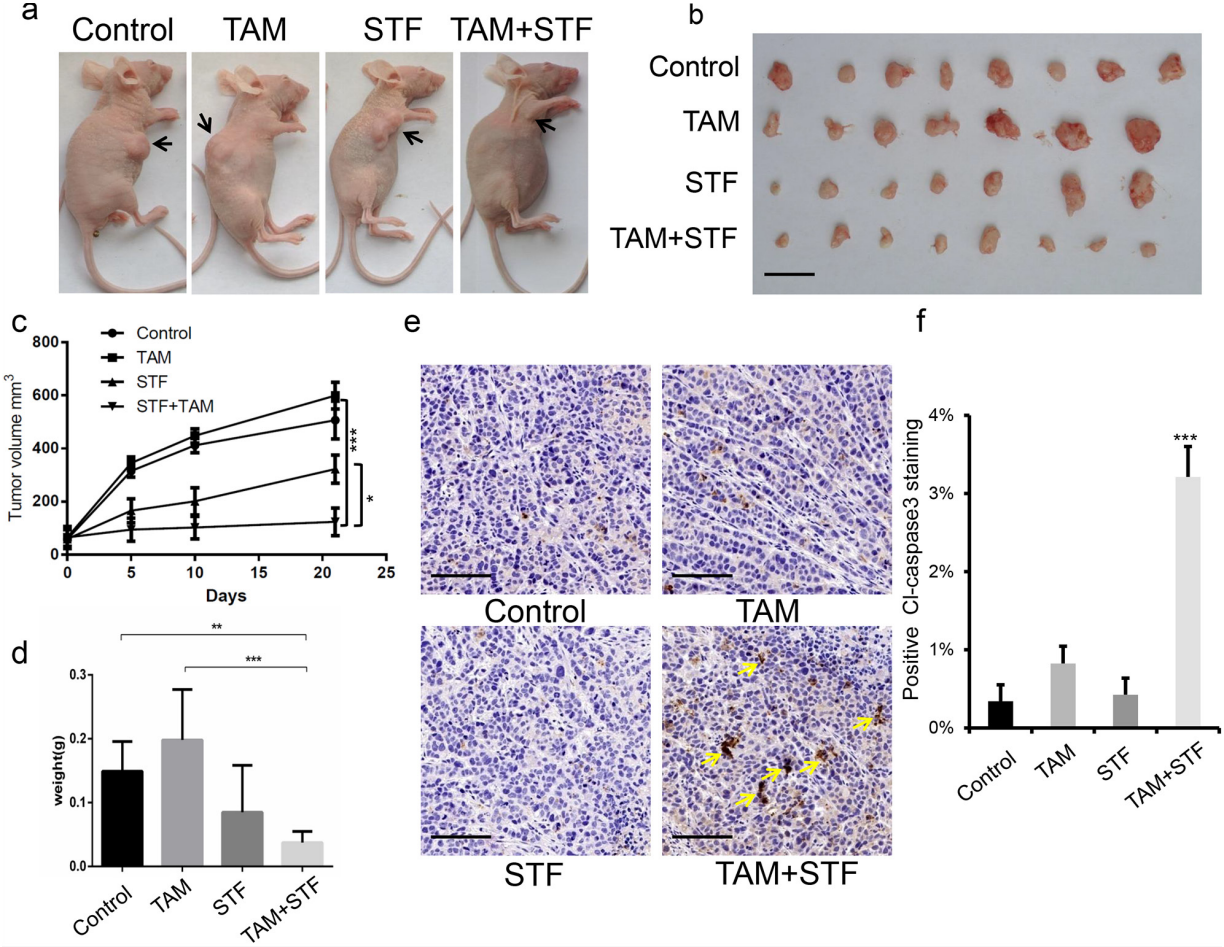
STF-080310 has a synergistic therapeutic effect with tamoxifen in a murine xenograft breast tumor model **a.** Representative images of nude mice 21 days after injected with MCF7-TAMR cells of control group; tamoxifen-treated group (TAM); STF-080310-treated group (STF) and tamoxifen- plus STF-080310-treated group (TAM+STF). **b.** At least 7 mice per group were sacrificed to analyze tumor progression. Compared to the other groups, the TAM+STF treated mice had significantly smaller tumors. Scale bars, 1 cm. **c.** Tumor growth curve of control group, TAM group, STF group and TAM+STF group. Tumors' sizes were determined 5 days, 10 days and 21 days after injecting MCF7-TAMR cells into nude mice. *P < 0.05; ***P < 0.001 (Wald's test). **d.** Tumor weights were measured and results are shown as mean (±s.e.m). The TAM+STF treatment significantly decreases tumor weight compare to control group and to the two single treatment groups, suggesting a synergistic effect of STF and TAM in tamoxifen-resistant breast tumor therapy *in vivo*. **P < 0.01; ***P < 0.001. (unpaired, two-sided *t*-test). **e.** Immunohistochemistry analysis of Caspase3 in breast tumor tissue in all 4 groups. Yellow arrows indicate positive Caspase3 staining. Scale bars, 50 μm. **f.** Positive Caspase3 staining of 4 treatment groups are shown as mean percentages (±s.e.m.) of (e). ***P < 0.001. (unpaired, two-sided *t*-test).

To evaluate whether STF-080310 can suppress tumorigenesis *in vivo*, we used a murine breast cancer xenograft model by injecting the MCF7-TAMR cells into the dorsal flank of female nude mice. When tumors reached approximately a size of 150 mm^3^, we divided the tumor-bearing mice into 4 different groups: control group (treated with DMSO); tamoxifen-treated group; STF-080310-treated group and tamoxifen plus STF-080310 treated group. Each group was comprised of at least 7 mice and all the mice were treated for 3 weeks (Figure 3a). When we isolated the tumors from the four groups, the tamoxifen plus STF-080310-treated mice had smaller tumors compared to the control group and to the single drug treatment groups (Figure 3b). Moreover, tumor growth curve indicated that tamoxifen plus STF-080310-treated mice showed significantly slower tumor progression compared to other 3 groups (Figure 3c), also the tumor weight from the dual treatment of tamoxifen and STF-080310 group were much less than other groups (Figure 3d). These results demonstrated that STF-080310 and tamoxifen had synergistic therapeutic effects on tamoxifen-resistant breast tumors *in vivo*. Furthermore, we investigated the pathological and proliferation status of these tumors by H&E and Ki67 staining, but no significant difference were detected among these groups (Supplementary Figure S2a, S2b), however, we found dramatically more Caspase3 positive staining in the tamoxifen and STF-080310 treated tumors, suggesting more apoptotic cell death of tumor cells in this group (Figure 3e, 3f). Finally, we analyzed XBP1s expression levels in all the breast tumors by immunohistochemistry and found that the XBP1s expression was significantly inhibited in both groups treated with STF-080310 alone or with STF080310 and tamoxifen. (Supplementary Figure S3), which indicated that STF-080310 could efficiently inhibit XBP1s' expression *in vivo*. Taken all together, STF-080310 can significantly reinstate tamoxifen sensitivity *in vivo* by inhibiting XBP1s function and thus can be used together with tamoxifen to efficiently delay breast tumor progression.

### Elevated XBP1s expression is highly correlated with poor survival in ER^+^ breast cancer patients

**Figure 4:**
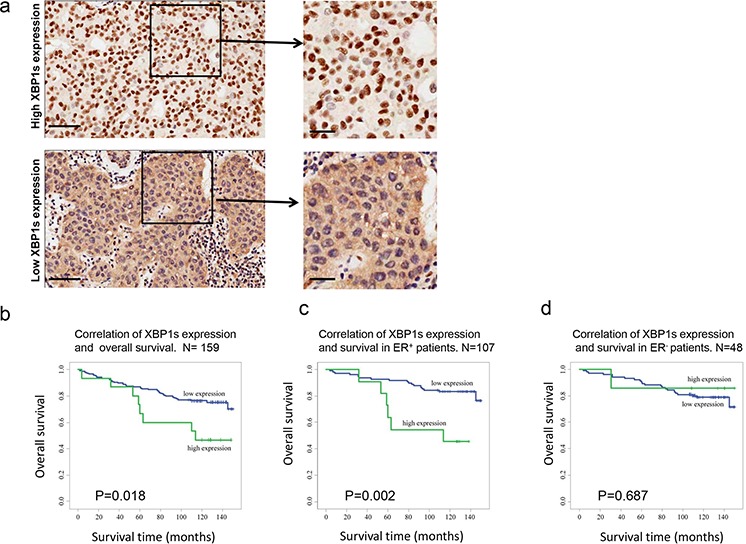
XBP1s expression is highly correlated with overall survival of ER+ breast cancer patients **a.** Representative images of high and low XBP1s expression level in clinical patients' samples. Scale bars, left panel, 100 μm, right panel, 20 μm. **b, c, d.** Correlation of XBP1s expression level and overall survival in all patients (b), ER^+^ patients (c) and ER^−^ patients (d). *P* < 0.05 is considered as significant.

Over 170 patients' samples were collected for immunohistochemistry analysis for XBP1s expression. The patients had been categorized into different sub groups according to their various characteristics. We established standard protocols to define high and low expression of XBP1s (see details in Materials and Method), and representative images of high and low XBP1s expression levels are shown in Figure 4a. Furthermore, we investigated the correlation between XBP1s expression and overall survival. The results indicated that for the high XBP1s expression patients, the survival ratio was only 46.7%. Conversely, for low XBP1s expression patients the survival ratio was 75%, which was significantly higher than high expression patients (Figure 4b). Moreover, we analyzed the correlation of XBP1s expression and survival both in ER^+^ and ER^−^ subgroups respectively, and found that XBP1s expression was only associated with overall survival in the ER^+^ group but not in the ER^−^ group (Figure 4c, 4d).

### XBP1 is not an independent prognostic factor in ER^+^ and ER^−^ groups

**Figure 5:**
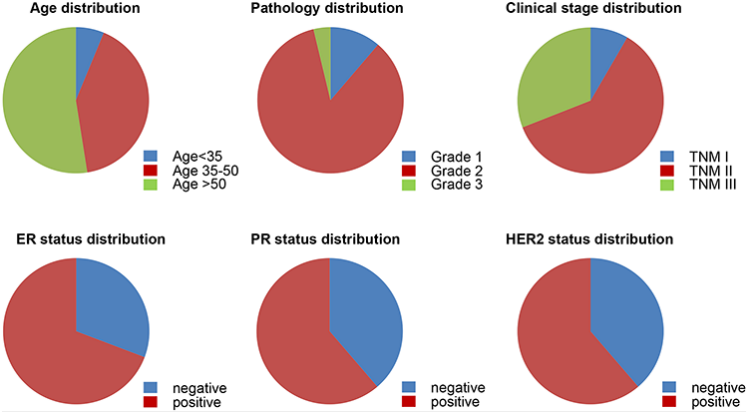
Characterization of patients Patients are categorized with respect to age, pathology grade, clinical stage classification, ER status, PR status and HER2 status.

All the patients were categorized according to their pathological grade, clinical stage, as well as their ER, PR and HER2 status (Figure 5). We analyzed different prognostic factors and found that only in ER^+^ groups did XBP1s expression as well as pathological levels, tumor size, T stage, N stage, TNM stage significantly affect overall survival. However, in the ER^−^ group, all these factors had no correlation with overall survival by Log-rank test (Table 1). Multivariate Cox regression analysis in ER^+^ and ER^−^ groups are displayed in Table 2. In the ER^+^ group, XBP1s expression had no statistically significant contribution to prognosis (*P* = 0.074) although the relative risk was obviously high (2.539, 95%CI 0.931–7.059). The only factor that had statistical significance in this group was tumor size (*P* = 0.039), with a RR of 2.943 (95%CI 1.056–8.200). However, none of these factors displayed statistically significance in prognostic predicting in the ER^−^ group. Overall, it is concluded that XBP1s expression cannot be used as an independent prognostic factor.

**Table 1:** Kaplan-meier survival analysis grouped by ER status respective to XBP1s expression and other clinicalpathologic parameters

		ER (+) subgroup				ER (−) subgroup			
			Censored				Censored		
		Total *N*	Survival *n*	Percent		Total *N*	Survival *n*	Percent	
					Log Rank *P*				Log Rank *P*
XBP1S	low	96	79	82.3%	0.002	44	26	59.1%	0.782
	high	11	5	45.5%		4	2	50.0%	
	Overall	107	84	78.5%		48	28	58.3%	
Age1	< 35	7	7	100.0%	0.227	3	1	33.3%	0.322
	35 − 50	44	36	81.8%		18	13	72.2%	
	> 50	56	41	73.2%		27	14	51.9%	
	Overall	107	84	78.5%		48	28	58.3%	
Age2	< 50	49	41	83.7%	0.235	18	11	61.1%	0.978
	≥ 50	58	43	74.1%		30	17	56.7%	
	Overall	107	84	78.5%		48	28	58.3%	
Pathology grade	I	14	10	71.4%	0.000	4	2	50.0%	0.808
	II	87	74	85.1%		44	26	59.1%	
	III	6	0	.0%		0	0	0	
	Overall	107	84	78.5%		48	28	58.3%	
Tumor size	< 2 cm	24	20	83.3%	0.001	3	3	100.0%	0.309
	2cm − 4cm	75	60	80.0%		32	16	50.0%	
	> 4 cm	6	3	50.0%		13	9	69.2%	
	Overall	105	83	79.0%		48	28	58.3%	
T stage	T1	24	20	83.3%	0.042	12	7	58.3%	0.996
	T2	75	60	80.0%		28	16	57.1%	
	T3	6	3	50.0%		8	5	62.5%	
	Overall	105	83	79.0%		48	28	58.3%	
N stage	N0	44	36	81.8%	0.016	16	9	56.3%	0.880
	N1	31	28	90.3%		16	10	62.5%	
	N2	24	14	58.3%		12	7	58.3%	
	N3	4	3	75.0%		4	2	50.0%	
	Overall	103	81	78.6%		48	28	58.3%	
TNM stage	TNM1	9	7	77.8%	0.012	4	4	100.0%	0.305
	TNM2	64	56	87.5%		26	14	53.8%	
	TNM3	29	18	62.1%		18	10	55.6%	
	Overall	102	81	79.4%		48	28	58.3%	
PR	−	18	13	72.2%	0.441	42	24	57.1%	0.447
	+	88	70	79.5%		4	3	75.0%	
	Overall	106	83	78.3%		46	27	58.7%	
HER-2	−	77	60	77.9%	0.935	26	14	53.8%	0.470
	+	30	24	80.0%		22	14	63.6%	
	Overall	107	84	78.5%		48	28	58.3%	
HER-2(FISH)	−	81	63	77.8%	0.923	28	14	50.0%	0.093
	+	14	11	78.6%		4	4	100.0%	
	Overall	95	74	77.9%		32	18	56.3%	

**Table 2:** Multivariate Cox regression analysis in ER^+^ and ER^−^ groups

	ER (+) subgroup						ER (−) subgroup						
					95.0% CI							95.0% CI	
	CC	SE	P	RR	lower	upper		CC	SE	P	RR	lower	upper
XBPISn	.932	.522	.074	2.539	.913	7.059	XBPISn	.730	.421	.083	2.074	.909	4.736
T stage	− .035	.608	.954	.966	.294	3.177	Pathology	.621	.537	.247	1.862	.650	5.331
N stage	.206	.442	.641	1.228	.517	2.920	N stage	− .079	.311	.799	.924	.502	1.701
TNM stage	.439	.802	.584	1.551	.322	7.466	TNM stage	.752	.529	.155	2.122	.752	5.990
Tumor size	1.079	.523	.039	2.943	1.056	8.200	ER status	− .409	.447	.360	.664	.276	1.596
Pathology	.937	.544	.085	2.551	.878	7.414	PR status	− .607	.450	.177	.545	.226	1.316

## DISCUSSION

Endocrine therapy is the most efficient systemic therapy for estrogen receptor positive breast cancer; however, many ER^+^ breast cancer patients can acquire resistance to endocrine therapy. Previous studies have shown that this resistance is associated with the UPR response and elevated signal transduction pathways such as the EGFR/MAPK, PI3K/AKT/mTOR pathways [15]. Alternative treatments include applying different endocrine drugs such as anastrozole and fulvestrant; or combinational endocrine therapy with small molecular inhibitors such as gefitinib, lapatinib and everolimus [16]. Clinical studies have shown that aromatase inhibitors (AIs) can significantly increase the therapeutic efficiency of postmenopausal breast cancer patients who have tamoxifen resistance. Even among those patients who got no therapeutic effects with AIs, 30% of them can benefit with subsequent usage of fulvestrant [17, 18]. It is also reported that mTOR inhibitors in combination with exemestane can improve progression-free survival in postmenopausal breast cancer patients. [15] *In vitro* studies show that mTOR inhibitors combined with tamoxifen can increase toxicity by killing up to 56% more ER^+^ breast tumor cells than the single drug treatment [19]. Despite these inspiring data, clinical trials evaluating the combination of endocrine therapy and trastuzumab or the TKIs, like gefitinib, erlotinib and lapatinib, have produced various results. [20, 21] Nevertheless, tamoxifen is still the most important endocrine therapy for premenopausal breast cancer patients, therefore it is important and urgent to investigate the mechanisms involved in breast tumor cell resistance to tamoxifen and to establish new treatment strategies.

Human X-box binding protein-1 (XBP1) is an alternatively spliced transcription factor that participates in a stress-signaling pathway to protect cells from damage. It has also been reported that XBP1 is involved in the unfolded protein response (UPR) and ER stress response under the control of IRE1. Clarke et.al found that over-expression of the spliced variant of the gene in estrogen receptor-positive breast cancer cells led to reduced sensitivity to tamoxifen and faslodex. Subsequent studies reported that XBP1s expression in ER^+^ breast tumors correlated with poor clinical responsiveness to tamoxifen, however, the underlying signaling mechanisms affected by XBP1s, as well as the effects of splicing on anti-estrogen resistance, remain unclear. It is hypothesized that XBP1s mediates anti-estrogen resistance in part through regulating nuclear factor kappa B (NF-kappaB) signaling. Overexpression of XBP1 in MCF7 and LCC1 anti-estrogen-sensitive breast cancer cells resulted primarily in an increase in XBP1s and an induction of RelA expression at both the mRNA and protein levels [22] Chen et.al reported that XBP1 was activated in triple negative breast cancer, a highly aggressive malignant subtype of breast cancer. They found that deletion of XBP1inhibited both tumor growth and tumor relapse as well as decreased the CD44^high^ CD24^low^ cell population by inhibiting the HIF pathway. [23]

In our study, we established a MCF7-TAMR cell line by long-term, low concentration exposure of MCF-7 cell to tamoxifen. Cell viability assays showed that the MCF7-TAMR cells' sensitivity to tamoxifen was reduced by 1.7 ~ 2.4 fold compare to control cells; this was also confirmed by morphological analysis. The method of low concentration tamoxifen exposure to induce cell resistance to tamoxifen is a reasonable and efficient way of studying drug resistance *in vitro*, which essentially mimics the real-life clinical situation drug resistance occurs, as most drug resistance is induced by long term drug usage.

We next investigated the expression levels of XBP1 and its upstream regulator, IRE1, in the MCF7-TAMR cell lines, and showed that the expression level of both XBP1s and p-IRE1 were increased significantly. Importantly, this enhanced expression occurred shortly after tamoxifen was given and in a concentration-dependent manner. Although there may be other pathways affected in the MCF7-TAMRcells, we conclude that the up-regulation of IRE1-XBP1 is at least partially associated with the reduced sensitivity to tamoxifen, and that the UPR and ER stress responses are activated in MCF7-TAMR cells. Indeed, we compared gene expression profiles between MCF7-TAMR cells and control cells using a next generation sequencing approach and a new bioinformatics model, the result showed 1215mRNA and 513 small RNA transcripts were changed which could be clustered into ER functions, cell cycle regulation, transcription/translation, and mitochondrial dysfunction.

To validate our findings, we next used a novel IRE1-XBP1 inhibitor, STF-083010, to treat the MCF7-TAMRcells. STF083010 is an inhibitor of IRE1α endonuclease activity; which can block endogenous XBP1 mRNA splicing and displays cytostatic and cytotoxic effects in CD138^+^ multiple myeloma (MM) cells *in vitro* [14], STF083010 is also reported to inhibit bortezomib-induced XBP1 activity in myeloma xenografts *in vivo* but does not alter IRE1α kinase activity [24]. Our *in vitro* experiments showed that STF-083010 could specifically inhibit XBP1 splicing and increased the sensitivity of the MCF7-TAMRcells to tamoxifen by up to 60%. We also injected MCF7-TAMRcells into nude mice to study STF-083010 function *in vivo*. Not surprisingly, the combinatory treatment of STF-083010 and tamoxifen could reduce tumor weight and tumor diameter by up to 75% and 38.3% respectively when compared to single-drug treatment groups. As described previously [13, 14], STF-083010is a novel IRE1-XBP1 inhibitor which inhibits the RNase activity of IRE1 but without affects its kinase functions, thereby specifically regulating downstreamXBP1s expression while greatly minimizing undesired side-effects. We believe that the decreased XBP1s expression level induced by STF-083010 somehow restored the tumor sensitivity to tamoxifen, however, we cannotexclude other factors which might contribute to this striking therapeutic result, and the detailed mechanism of how STF-083010 re-establishes the sensitivity of resistant tumors to tamoxifen *in vivo* needs to be further investigated.

As XBP1s has been implied to play a key role in drug resistance in certain subtypes of breast cancers [23], we systematically reviewed breast cancer patients' samples regarding XBP1s expression. Intriguingly, we found thatXBP1s expression was correlated with overall survival not only in the ER^+^ patients, but also among all breast cancer patients when we pooled them together. Our data demonstrates that high XBP1s expression levels lead to poor survival after endocrine therapy, which suggests that XBP1s could be considered as a prognosis factor and contribute to drug resistance in the clinic. Therefore, small molecular inhibitors such as STF083010, which targets IRE1-XBP1, may have potential therapeutic effects for endocrine therapy resistant patients.

In essence, IRE1-XBP1 plays an essential role in the UPR and ER stress responses, which have been implicated in the development of drug resistance. Our data suggests that a combination of IRE1-XBP1 inhibitors and endocrine therapy would be an alternative strategy for breast cancer therapy in the future.

## MATERIALS AND METHODS

### MCF-7 cell cultures, T47D cell cultures and establishment of tamoxifen-resistant cell lines

The original MCF-7 and T47D breast cancer cells were purchased from ATCC. Both cells were cultured in DMEM supplemented with 10% fetal bovine serum (Gibco, Life Technology) and 1% penicillin-streptomycin (standard medium) at 37°C and 5% CO2. To develop tamoxifen-resistant cells, the MCF-7 cells were cultured in the same conditions supplemented with 1 μM 4-OH tamoxifen (Sigma Aldrich) for 30 days and the T47D cells were incubated with 10 μM 4-OH tamoxifen for 30 days respectively.

### Quantitative RT-PCR

Total RNA was prepared from control and tamoxifen-resistant MCF-7 cells using the RNeasy Mini Kit (Qiagen) in accordance with the manufacturer's instructions. Total RNA (1 μg) was subjected to reverse transcription using random hexamers (Roche) and SuperscriptII (Invitrogen) followed by quantitative PCR analysis. The following primers were used:

XBP1 spliced: 5′-GGTCTGCTGAGTCCGCAGCAGG-3′ (forward) and 5′-GGGCTTGGTATATATGTGG-3′ (reverse)
XBP1 total (XBP1 spliced+XBP1 unspliced): 5′-CGGAAGCCAAGGGGAATGAA-3′ (forward) and 5′-GTCCAGAATGCCCAACAGGA-3′ (reverse)
GAPDH: 5′-GAAGGTGAAGGTCGGAGTC-3′ (forward) and 5′-GAAGATGGTGATGGGATTTC-3′ (reverse).

### Western blot analysis

Approximately 2 × 10^6^ cells of each cell line were prepared overnight in 6-well plates. The next day, the supernatant was discarded and the cells were washed twice with PBS. Next the cells were lysed with appropriate RIPA buffer containing 1%PMSF, placed 20 min on ice and scraped using cell scraper. After 20 min incubation, cells/lysates were collected and transferred into tubes and centrifuged at 12,000g at 4°C for 10 min. The supernatant was pipetted into new tubes and the protein concentrations were determined by the BCA method (Thermo Scientific). Equal amount of protein (40 ug) of lysates were used for electrophoresis using Nu-Page 10% Bis-Tris gels and blotted onto nitrocellulose membranes. The membranes were washed briefly in TBS/0.1% Tween-20 (TBST), pH 7.4, and blocked in 5% milk or 5% BSA (diluted in TBST) for 1 h. The membranes were thenincubated with the following antibodies: XBP1s primary antibody (1:500; Abcam, 198999), XBP1u (1:1000, Abcam, abab37152), p-IRE1 (1:1000; Abcam, ab104157), IRE1 (1:1000; Cell Signaling, #3294), p-AKT (1:1000; Cell signaling, #9611), AKT (1:4000; Cell Signaling,#2944), Caspase3 (1:2000, Cell signaling,#9665), GADPH (1:5000; Cell Signaling, #5174) diluted in blocking solution overnight. Blots were then washed with TBST for 5 min three times and incubated in corresponding secondary antibody (1:20000; Cell Signaling, #7071, #7072) for 1 h. The membranes were processed with ECL-plus (Pierce, 32132) and then exposed on films in a dark room and the films were scanned by software Quantity One (Bio-Rad) and quantified by densitometry.

### Cell viability assay

Cell viabilities were determined by the MTT assay. Cells were seeded into 96-well culture plates with 0.5% fetus serum medium and started to adhere after 2–4 hours. Then the cells were treated with different conditions. After the treatment, cells were incubated with MTT working solution (Roche). The cell viabilities were determined by acquiring the OD value at 540 nm after incubation 4–6 hours in MTT. The relative viability was determined by OD value of experiment group/control group × 100%.

### Immunohistochemistry

Immunohistochemical analysis for XBP1s in breast tumor mice or patients was performed in a standard protocol. Briefly, 2 μm sections were obtained from formalin fixed and paraffin embedded tumor samples, sections were dehydrated and antigenic epitopes retrieved using a 10-mM citrate buffer and microwaving for 10 minutes. Specimens were stained with XBP1s antibody (1:100, abcam, ab37152), primary antibody staining was detected by peroxidase conjugated anti-rabbit IgG according to manufacturer's instructions (Roche). The ER, PR, and Her-2/neu staining were previously performed and recorded after initial surgery was done. The immunohistochemical expression of XBP1s was examined by light microscopy through calculating 1000 cells per 5 sights. The percentage of positive cells, as the extent of immunostaining, was quantified under the microscope and classified into five groups. 0: < 5% positive cells; 1: 5% to 24% positive cells; 2: 25% to 49% positive cells; 3: 50% to 74% positive cells and 4: > 75% positive cells. Intensity was scored as 0 for absence of staining, 1 for weak, 2 for moderate, and 3 for strong staining. The score of the intensity plus the percentage of positive staining were used to define expression levels. 0–1: negative; 2–3: little positive (+1); 4–5: moderately positive (+2); 6–7: strongly positive (+3). Lastly, a total score calculated by the product of staining intensity and positive percentage was used to divide all specimens into two groups: a low-expression group (0–5 scores) and a high-expression group (6–12 scores). Immunhistochemical staining was done using an automatic staining machine (Leica Bond3) or manually processed. Sections were dehydrated and antigenic epitopes were retrieved using a 10-mM citrate buffer and microwaving for 10 min. Specimen were then incubated with rabbit anti-Ki67 (Novocastra, 1:200), anti-cleaved caspase-3 (Cell Signaling, 9661, 1:200). Primary Ab staining was detected by peroxidase conjugated anti-rabbit IgG (DAKO, P0448, 1:500). Positive cells were counted on 20 randomly chosen tumor areas at ×400 magnifications in a double blinded fashion.

### Tumor growth model in nude mice

All mice were maintained according to the ethical animal license protocol complying with the Chinese law, and all animal studies were approved by the Huazhong Science and Technology University (HUST) Institutional Animal Care and Use Committee. Briefly, 4 * 10^6^ tamoxifen-resistant MCF-7 cells were injected subcutaneously into the flankof 4-week-old female nude mice (Animal experiment center of Tongji Medical College, HUST, WuHan, China). 4 days later, all the mice were divided into four groups randomly and treated with: 1) DMSO, 2) TAM 100ug/kg per day, 3) STF-083010 30mg/kg per week, 4) TAM combined with STF-083010 by intraperitoneal injection. Three weeks after injection, animals were killed and the four groups of tumors were examined. Tumor growth was evaluated by measuring tumor weight.

### Patients and samples

A total of 170 invasive breast cancer patients who underwent surgery at hospitalsof Jiangsu, Zhejiang and Shanghai that cooperated with National Engineering Center for Biochip at Shanghai during 2001–2004 were selected. 164 patients remained when six were discarded from the analysis due to lack of follow-up or lack of data. The age of patients ranged from 29 to 82 years old. Clinic-pathological characteristics are presented in Table 1. ER or PR-positive breast cancers were supposed to use tamoxifen as endocrine therapy under standard treatment.

### Statistical analyses

All statistical analyses were performed using SPSS16.0 software and the detail methods were indicated in figure legend. Survival curves were calculated using the Kaplan-Meier method, with the significance evaluated using the Mantel-Cox long-rank test. The prognostic significance of the parameters was assessed using the Cox proportional hazards model with overall survival as an end point. A multivariate analysis was performed using Cox model; previously identified prognostic factors in breast cancer were included in the model. The relations of XBP1s expression and clinic-pathological parameters were measured using the nonparametric KrusKal-Wallis and Mann-Whitney methods, using Spearman's correlation analysis. *p* < 0.05 was considered statistically significant. Correlations with numerical variables were analyzed by Mann-Whitney *U* test.

## SUPPLEMENTARY FIGURES


