# Supplementary material for: A novel chemical, STF-083010, reverses tamoxifen-related drug resistance in breast cancer by inhibiting IRE1/XBP1
Source: Oncotarget. 2015 Oct 19;6(38):40692–703. doi: 10.18632/oncotarget.5827 (PMC4747362; doi:10.18632/oncotarget.5827)
Supplement: Supplementary file 1 [file oncotarget-06-40692-s001.pdf]

SUPPLEMENTARY FIGURES

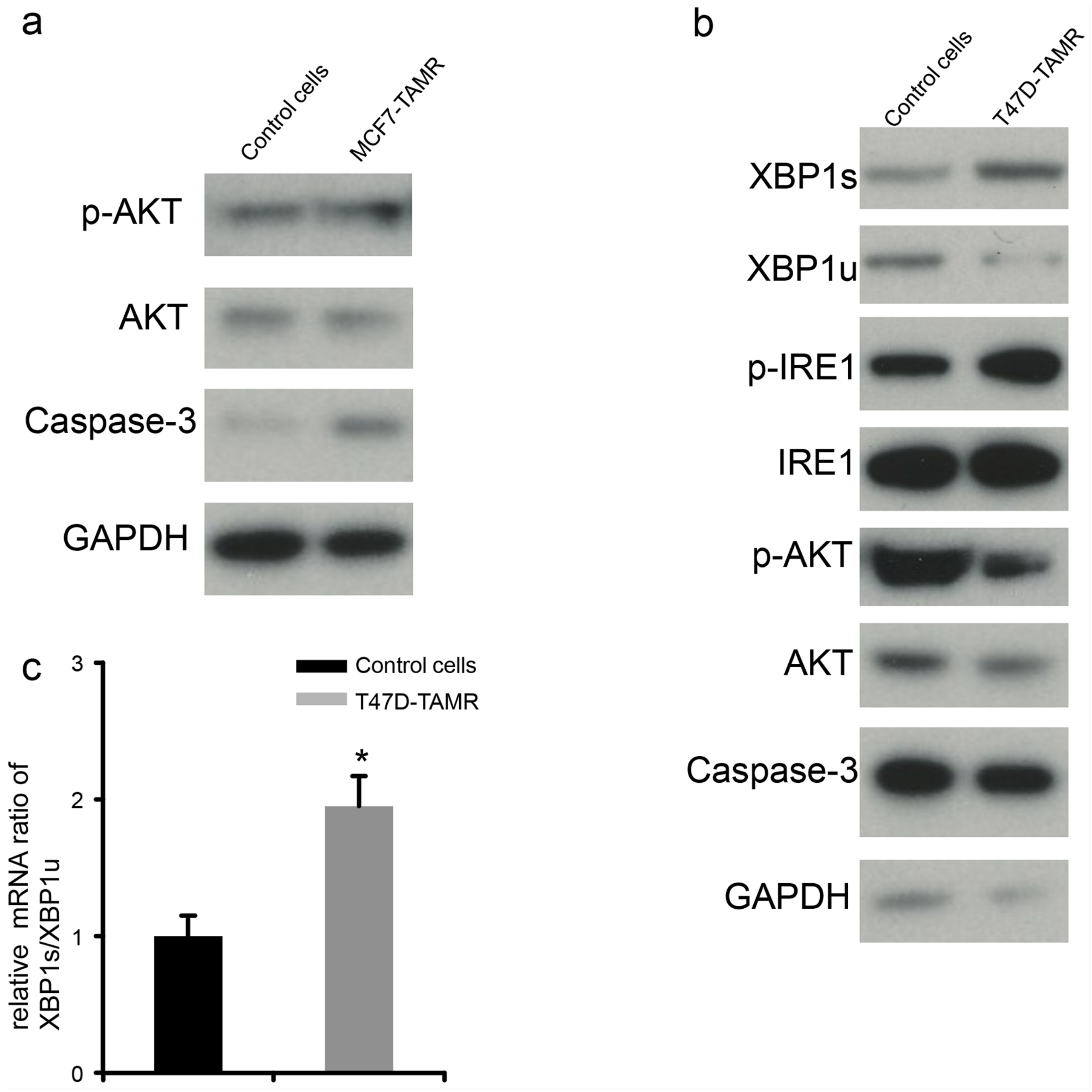

Supplementary Figure S1: a. Immunoblot showing no difference of AKT activity between control and MCF7-TAMR cells. Total Caspase3 level in MCF7-TAMR cells was higher compared to control cells, suggesting less apoptosis in MCF7-TAMR cells, however, no cleaved Caspase3 signal were detected in both groups. AKT and GAPDH were used as loading control. **b.** mRNA level of XBP1s/XBP1u ratio is determined by RT-PCR in both control and T47D-TAMR cells. The ratio in T47D-TAMR cells is significantly higher compared to control cells. *$P < 0.05$. (unpaired, two-sided *t*-test). **c.** Immunoblot analysis of XBP1s and p-IRE1 protein levels in control and T47D-TAMR cells. Representative results are shown. Note the XBP1s and phosphorylated IRE1expression level in T47D-TAMR cells are significantly higher than control cells, no difference was detected in Phospho-AKT and Caspase3 activity. The XBP1u, IRE1, AKT and GAPDH were used as loading control.

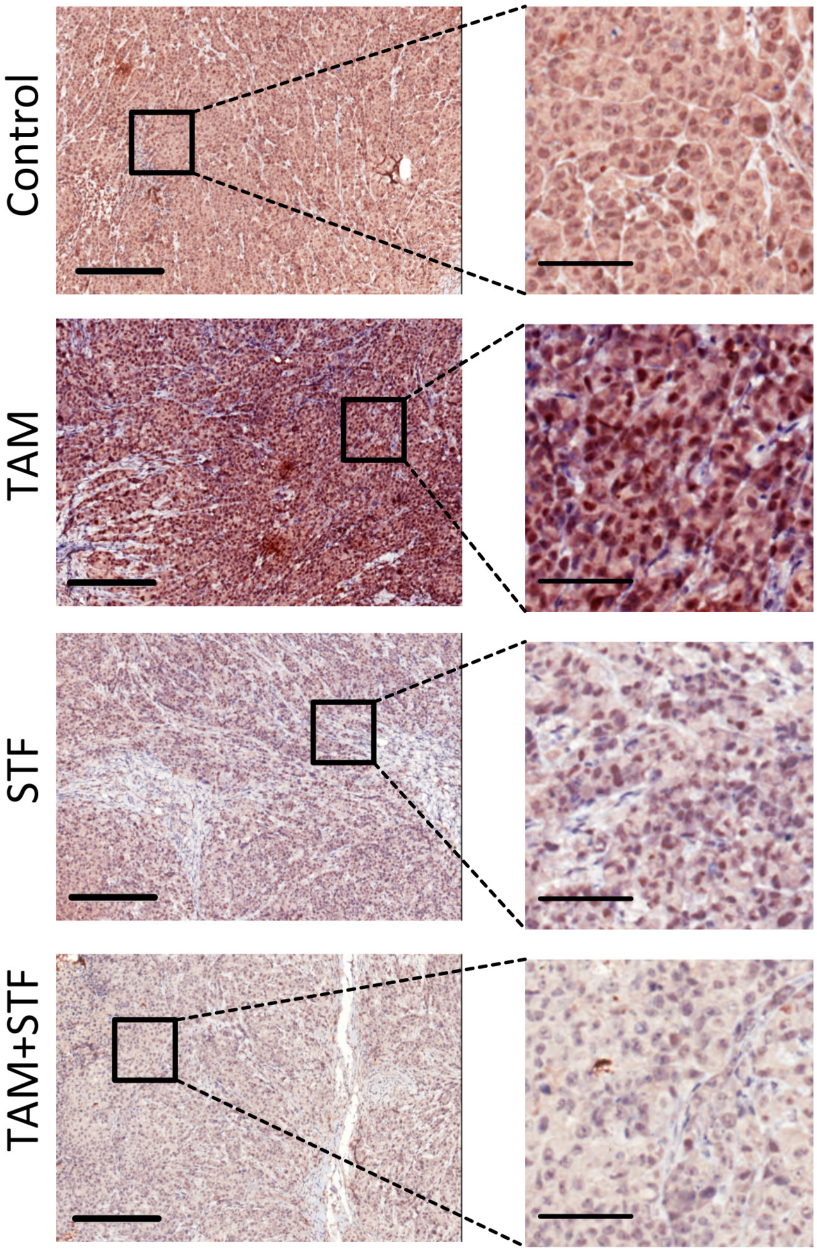

Supplementary Figure S2: Immunohistochemistry analysis of XBP1s expression in xenograft breast tumor tissues of control, TAM, STF and TAM+STF treatment groups. Selective images are shown and suggesting that in those two STF treatment groups, the XBP1s expression is efficiently inhibited. Scale bars, left panel, 200 µm, right panel, 50 µm.

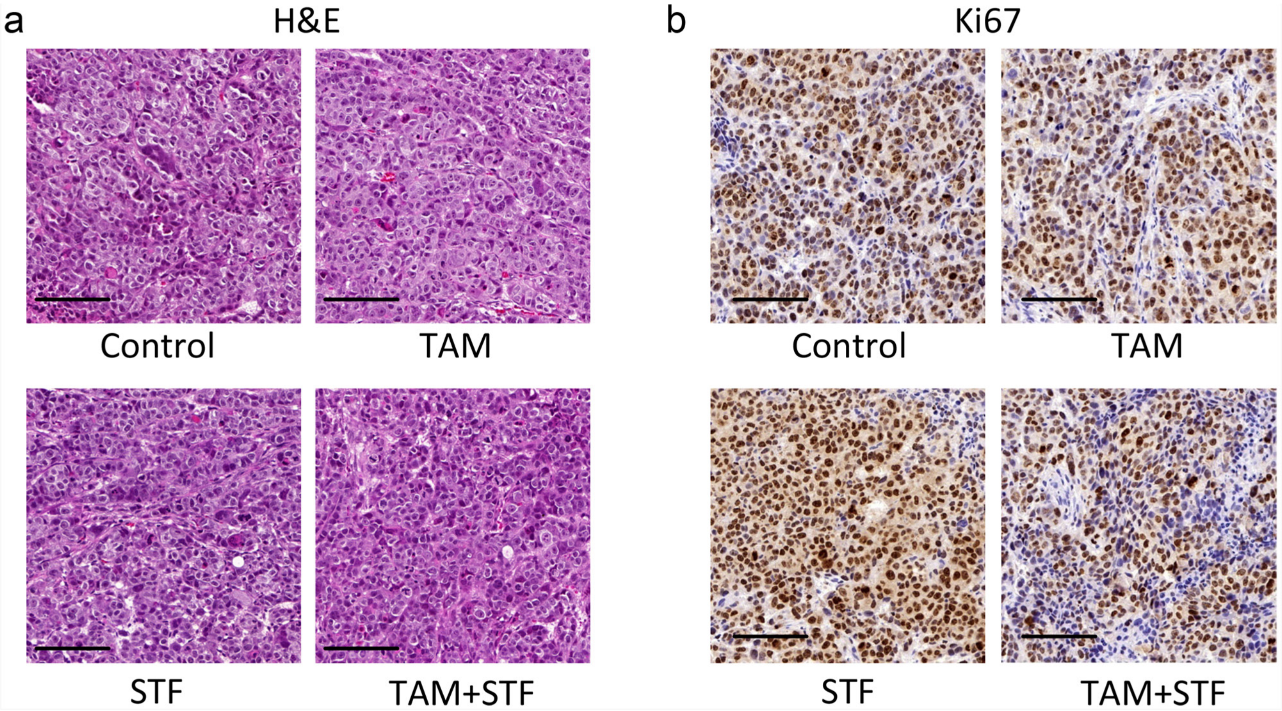

Supplementary Figure S3: a. Selective images of Hematoxylin and eosin stain in xenograft breast tumor tissues of control, TAM, STF and TAM+STF treatment groups. Scale bars, 100 μm. **b.** Selective images of Ki67 stainin breast tumor tissues of control, TAM, STF and TAM+STF treatment groups. Scale bars, 100 μm.
